# Supplementary material for: Genomics reveals heterogeneous Plasmodium falciparum transmission and selection signals in Zambia
Source: Commun Med (Lond). 2024 Apr 6;4:67. doi: 10.1038/s43856-024-00498-8 (PMC10998850; doi:10.1038/s43856-024-00498-8)
Supplement: Supplementary file 2 — Supplementary Information [file 43856_2024_498_MOESM2_ESM.pdf]

Supplementary Information for

# Genomics reveals heterogeneous *Plasmodium falciparum* transmission and selection signals in Zambia.

Abebe A. Fola[1#], Qixin He[1#], Shaojun Xie[2], Jyothi Thimmapuram[2], Ketaki P. Bhide[2], Jack Dorman[1], Ilinca I. Ciubotariu[1], Mulenga C. Mwenda[3], Brenda Mambwe[3], Conceptor Mulube[3], Moonga Hawela[3], Douglas E. Norris[4], William J. Moss[4,5], Daniel J. Bridges[6], Giovanna Carpi[1,4,7§]

[1] Department of Biological Sciences, Purdue University, West Lafayette, IN, USA
[2] Bioinformatics Core, Purdue University, Purdue University, West Lafayette, IN, USA
[3] PATH-MACEPA, National Malaria Elimination Centre, Lusaka, Zambia
[4] The Johns Hopkins Malaria Research Institute, W. Harry Feinstone Department of Molecular Microbiology and Immunology, Johns Hopkins Bloomberg School of Public Health, Baltimore, MD, USA
[5] Department of Epidemiology, Johns Hopkins Bloomberg School of Public Health, Baltimore, MD, USA
[6] PATH, Lusaka, Zambia
[7] Purdue Institute for Inflammation, Immunology, & Infectious Disease, Purdue University, West Lafayette, IN, USA

[§] Corresponding author: Giovanna Carpi, E-mail: gcarpi@purdue.edu

[#] These authors contributed equally to this work.

## SI Appendix includes:

- Supplementary Figures 1 to 14.

**Supplementary Figures**

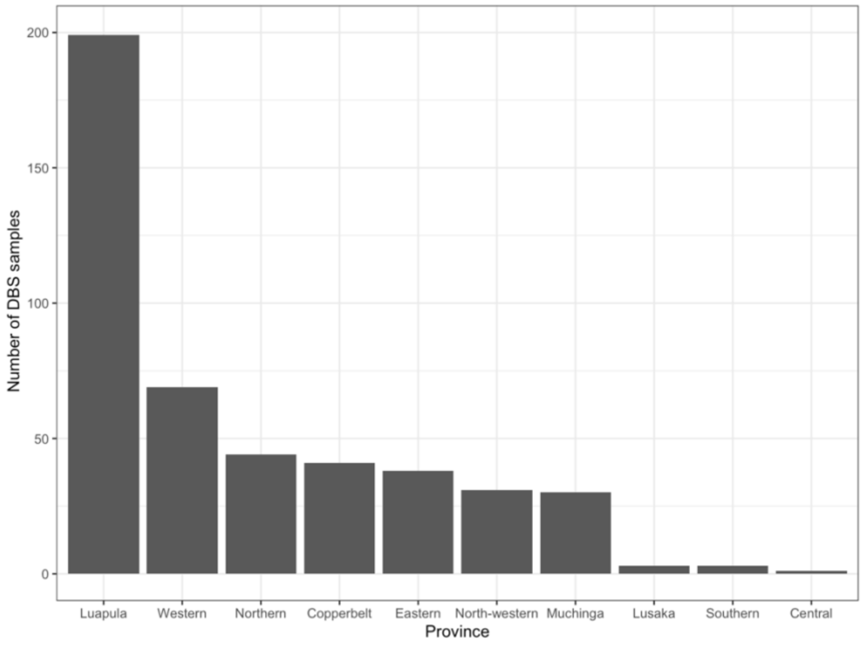


**Supplementary Figure 1. Distribution of WGS sequenced *P. falciparum* samples per province across Zambia.** A total of 459 (114 capture reactions-4plex and 1 capture reaction-3-plex) samples were sequenced from 10 provinces. Number of sequenced samples between provinces varied as a function of sampling efforts (see Methods for details).

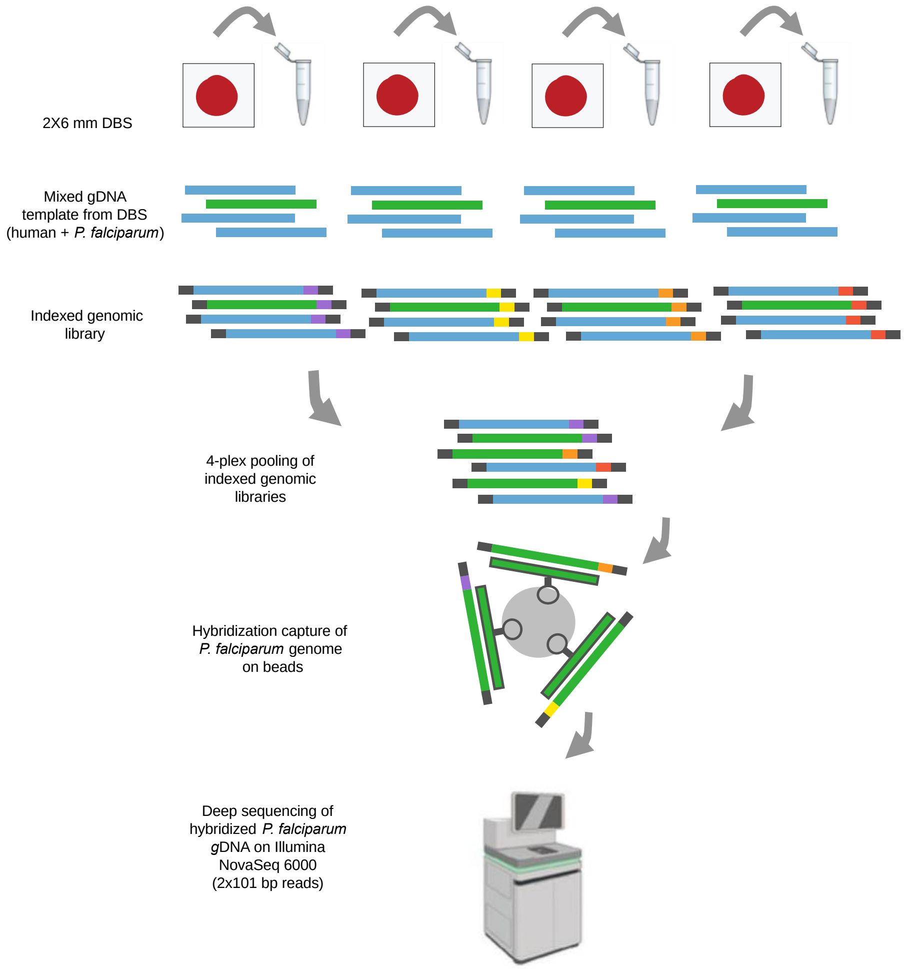


**Supplementary Figure 2. Multiplexed *P. falciparum* whole-genome capture and sequencing from DBS samples.** Schematic representation of 4-plex *P. falciparum* whole-genome capture and deep sequencing. Dual-Indexed genomic libraries were created from gDNA extracts from human DBS *P. falciparum* positive samples (mixed DNA template in blue and green colors), pooled in 4-plex and subsequently incubated with custom probes (SeqCap EZ). *P. falciparum* custom probes were designed to tile 98% *P. falciparum* 3D7 reference genome to enable target enrichment of *P. falciparum* DNA without amplification biases. Above, *P. falciparum* DNA (Green) hybridized to the probes while non-targeted DNA (Blue) is washed off. Hybridized DNA is then deep sequenced on Illumina NovaSeq 6000 platform.

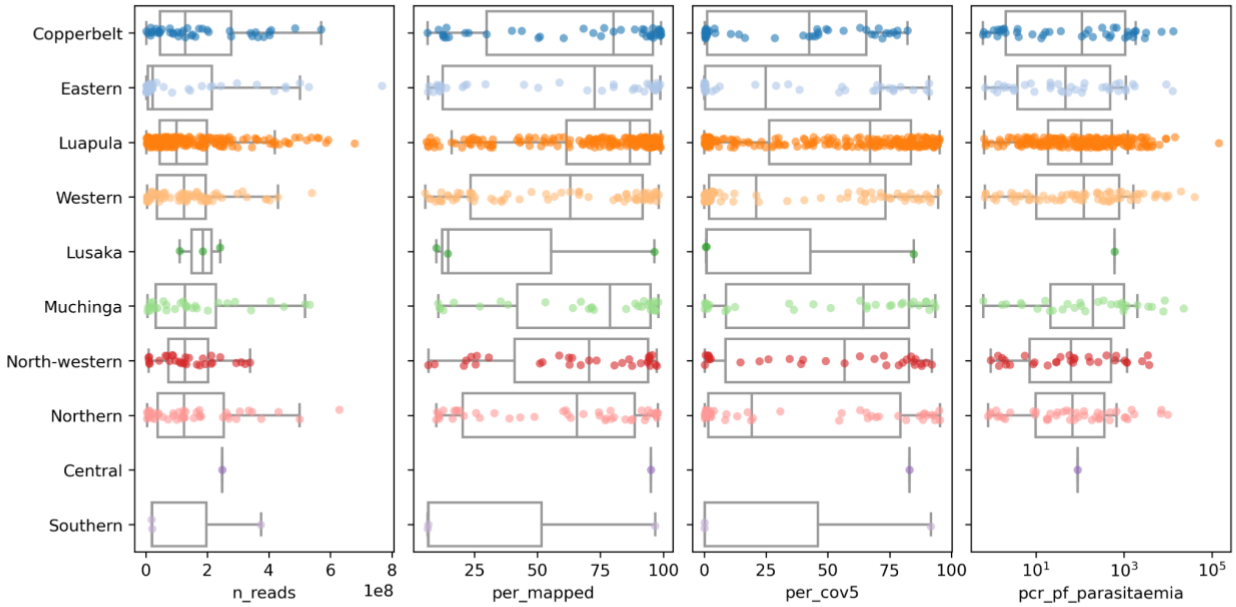


**Supplementary Figure 3. Sequencing metrics across 459 *P. falciparum* WGS sequenced samples by geographic origin.** From left to right, in the boxplots the x-axes represent the number of sequenced reads, the percentage of read mapping to *P. falciparum* 3D7 reference genome, the percentage of genome with minimum read depth of 5X, and the parasitemia estimated by PET-PCR (parasites/$\mu$L, log scale). The y-axis represents the geographic origin of the DBS samples at the provincial level. For each boxplot the middle line represents the median value of metrics of interest, the box represents the interquartile range, and the whiskers represent the range excluding outliers. Five samples (2 samples out of 3 from Lusaka Province and all 3 samples from Southern Province) were malaria positive by RDT but lacked an estimated parasitemia by PET-PCR, therefore these samples were omitted from the last bloxplot on the right.

**A.**

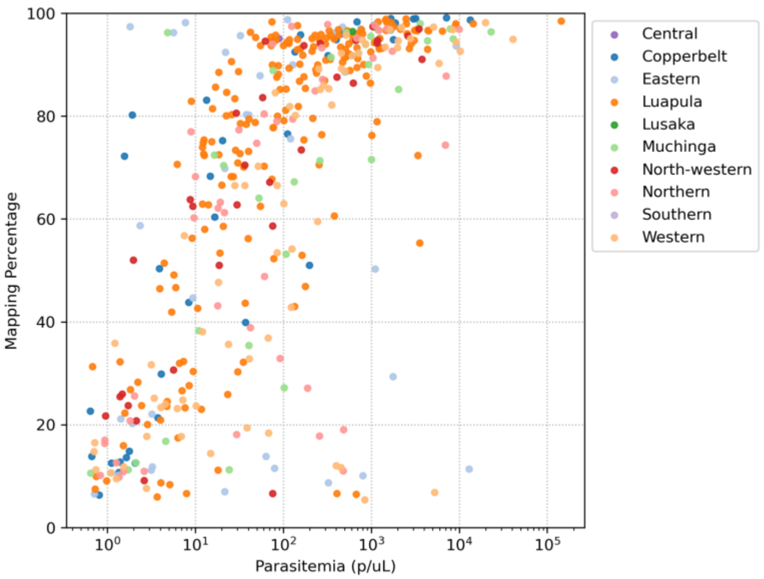


**B.**

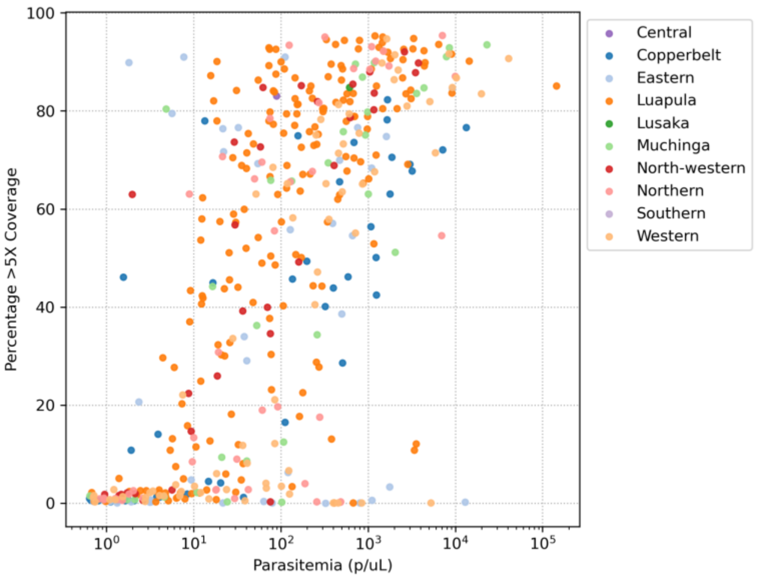


**Supplementary Figure 4. Correlates of *P. falciparum* capture efficiency and genome coverage**. WGS samples are represented by dots and colors denote sample origin **(A)** *P. falciparum* capture efficiency (the percentage of read mapping to *P. falciparum* 3D7 reference genome) is plotted against the estimated parasitemia (measured by PET-PCR). **(B)** The percentage of *P. falciparum* genome coverage with a minimum of 5X read coverage is plotted against the estimated parasitemia. *P. falciparum* parasitemia is a significant predictor of capture efficiency and genome coverage in univariate quasi-Poisson models.

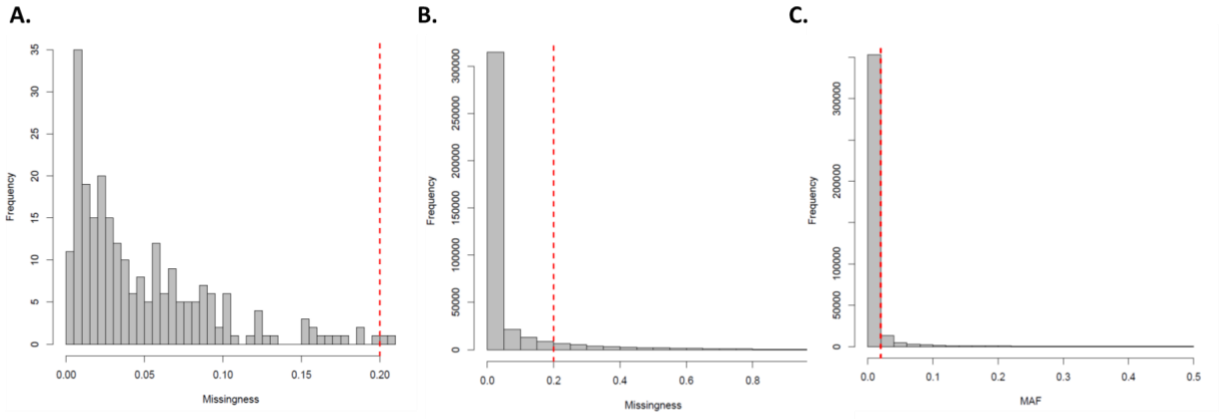


**Supplementary Figure 5. Distribution and filtering of sample missingness (A), SNP missingness (B), and minor allele frequency (MAF) (C).** Prior to variant filtering,121,403 SNPs with a VQSLOD >0 were identified across the 241 parasite genomes. After excluding variants in telomeric regions, a total of 389,097 biallelic SNPs were retained across the *P. falciparum* core genome. The vertical dashed red line in each plot represents the thresholds (0.2 sample and SNP missingness, and 0.02 MAF) used to omit samples and variants in the data that had a high degree of missingness. Following 0.2 SNP missingness filtering, 358,260 SNPs remained, and after MAF filtering at 0.02, 29,992 genome-wide biallelic SNPs were retained.

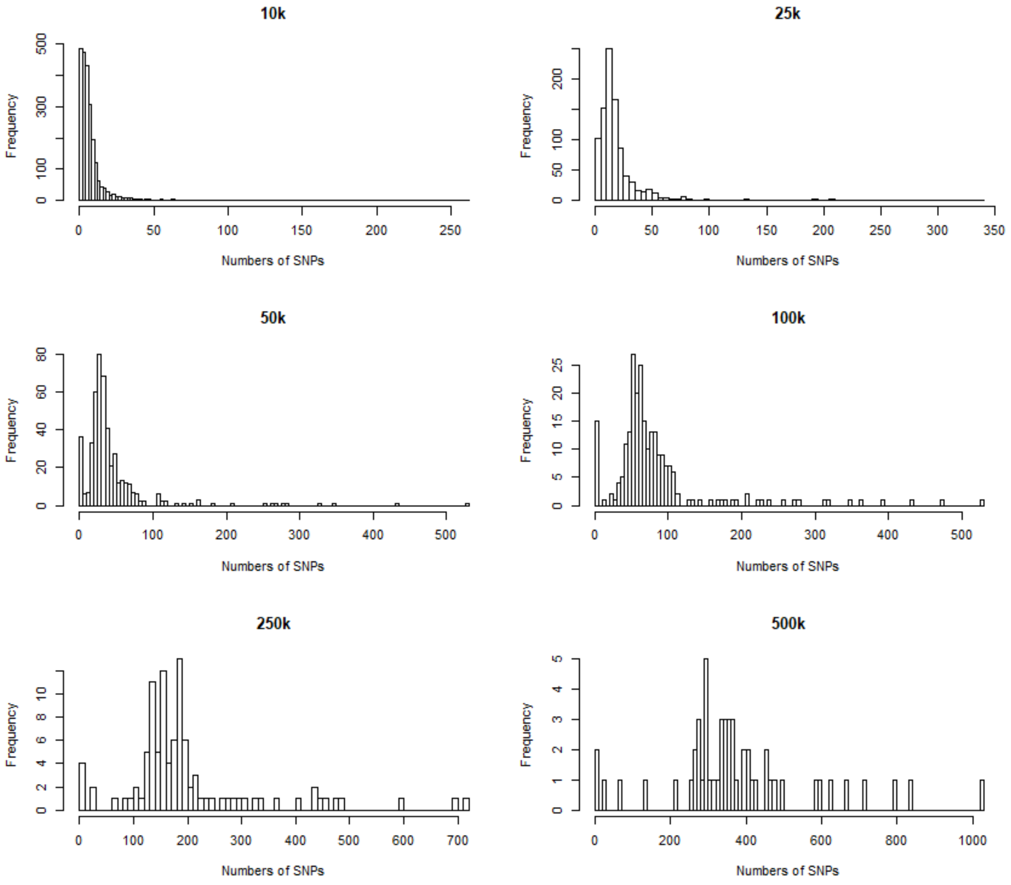


**Supplementary Figure 6. Distribution of 29,992 genome-wide SNPs using different window sizes.** Each SNP frequency plot illustrates a different window size from 10K to 500Kb. The x-axis indicates number of SNPs and y-axis indicates frequency of SNPs across sequenced samples.

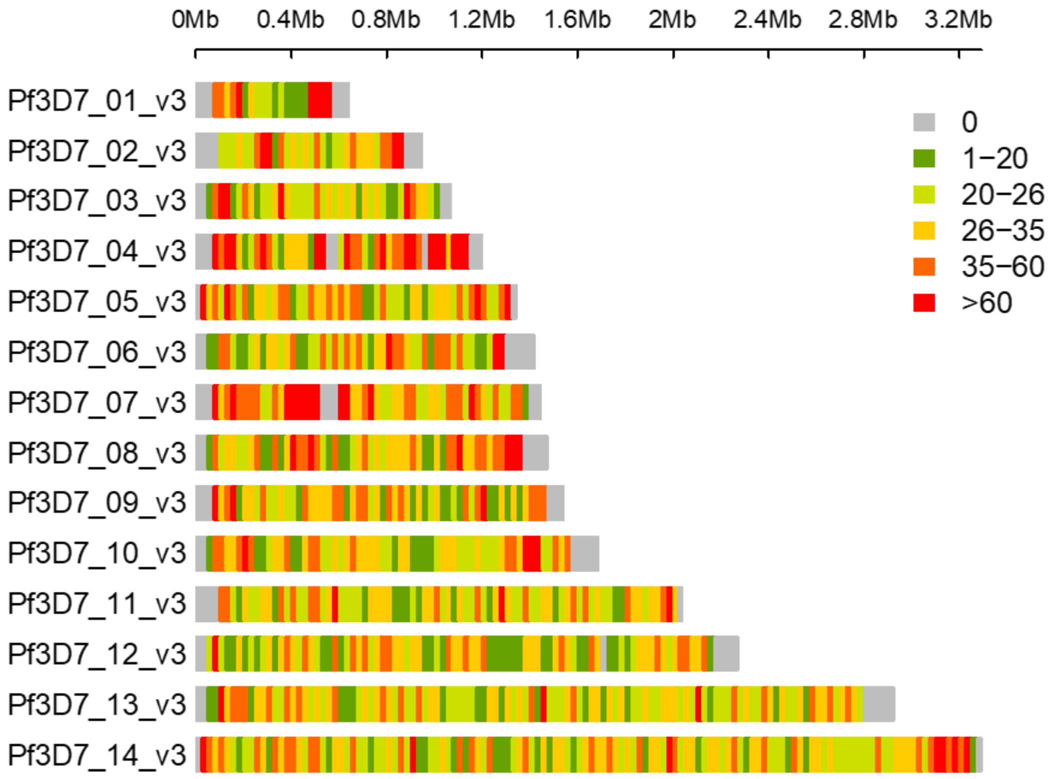


**Supplementary Figure 7. Genome-wide SNP density plot.** This figure illustrates the chromosome-wise distribution of SNP density, calculated within a 25Kb window size. The horizontal axis represents the chromosome length in megabases (Mb), while colors indicate varying levels of SNP density, ranging from low (depicted in green) to high (depicted in red). Masked telomeric and hypervariable regions are shown in gray.

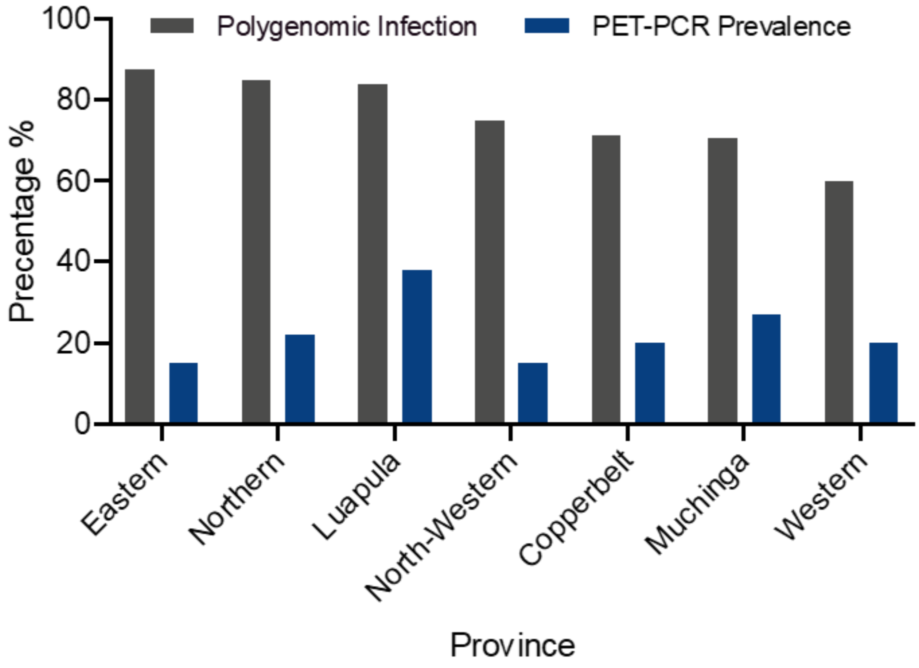


**Supplementary Figure 8. *P. falciparum* malaria prevalence and polygenomic infections per province.** The x-axis indicates provinces and y-axis indicate percentage of polygenomic infections (grey bars) (number of samples harboring more than one distinct parasite genome, $F_{WS} < 0.95$, divided by total number of samples sequenced per province X100). Blue bars represent *P. falciparum* malaria prevalence as estimated by PET-PCR.

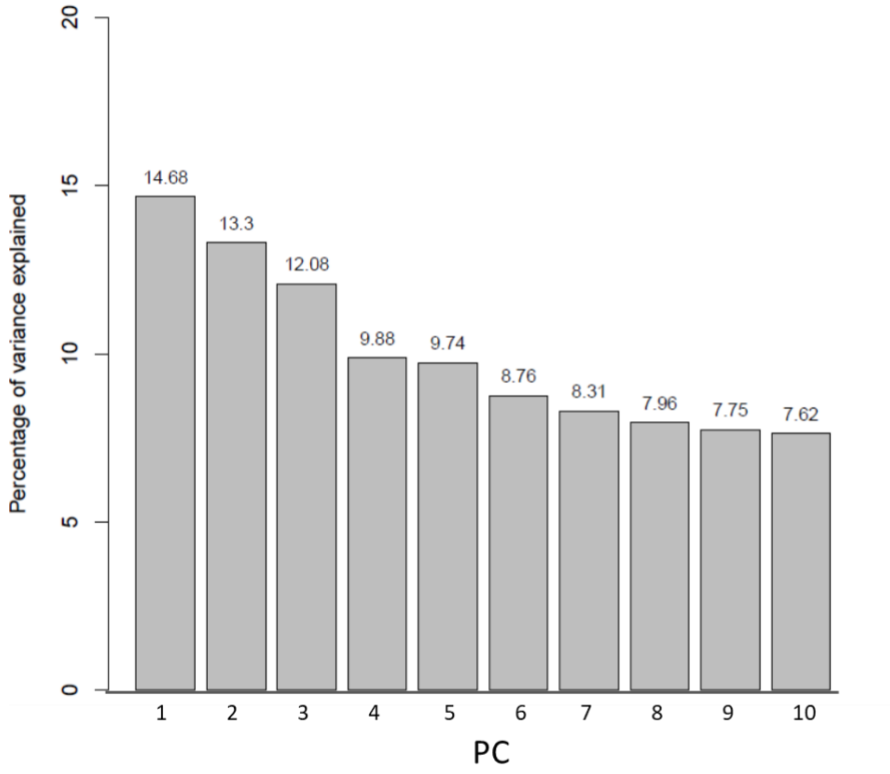


**Supplementary Figure 9. Percentage of variance explained by the Principal Components (PC) for Zambian *P. falciparum* parasites**. Bar plot shows the first 10 PCs and the percentage of variance explained by each.

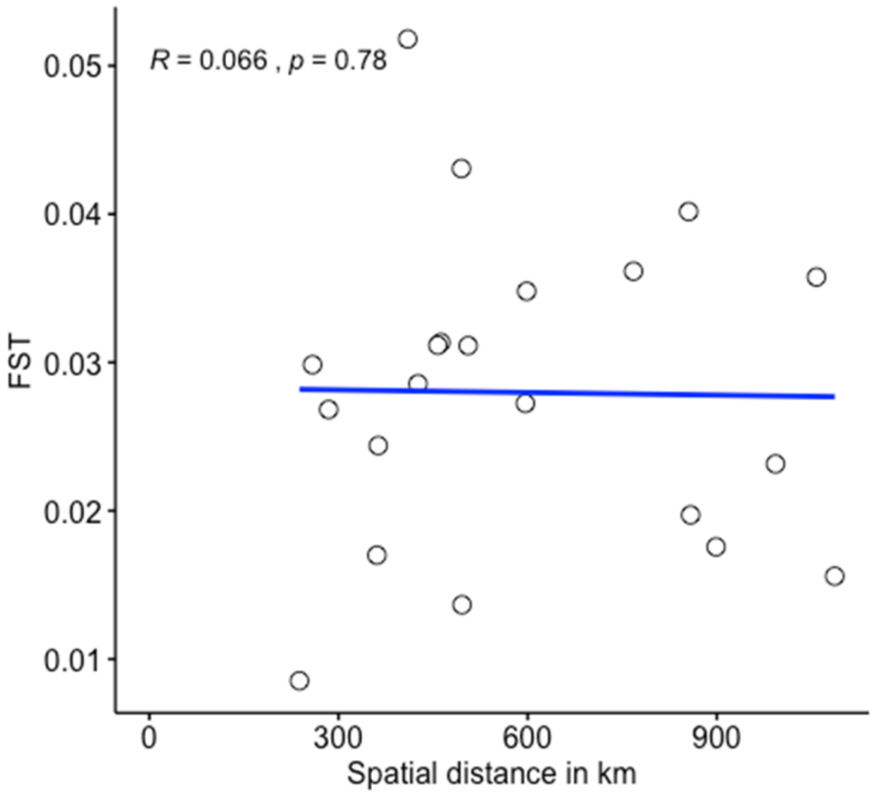


**Supplementary Figure 10. Mantel test showing the relationship between genetic distance and geographical distance.** Pairwise $F_{ST}$ value (genetic differentiation) and actual geographic distance between seven provinces across Zambia used for this analysis.

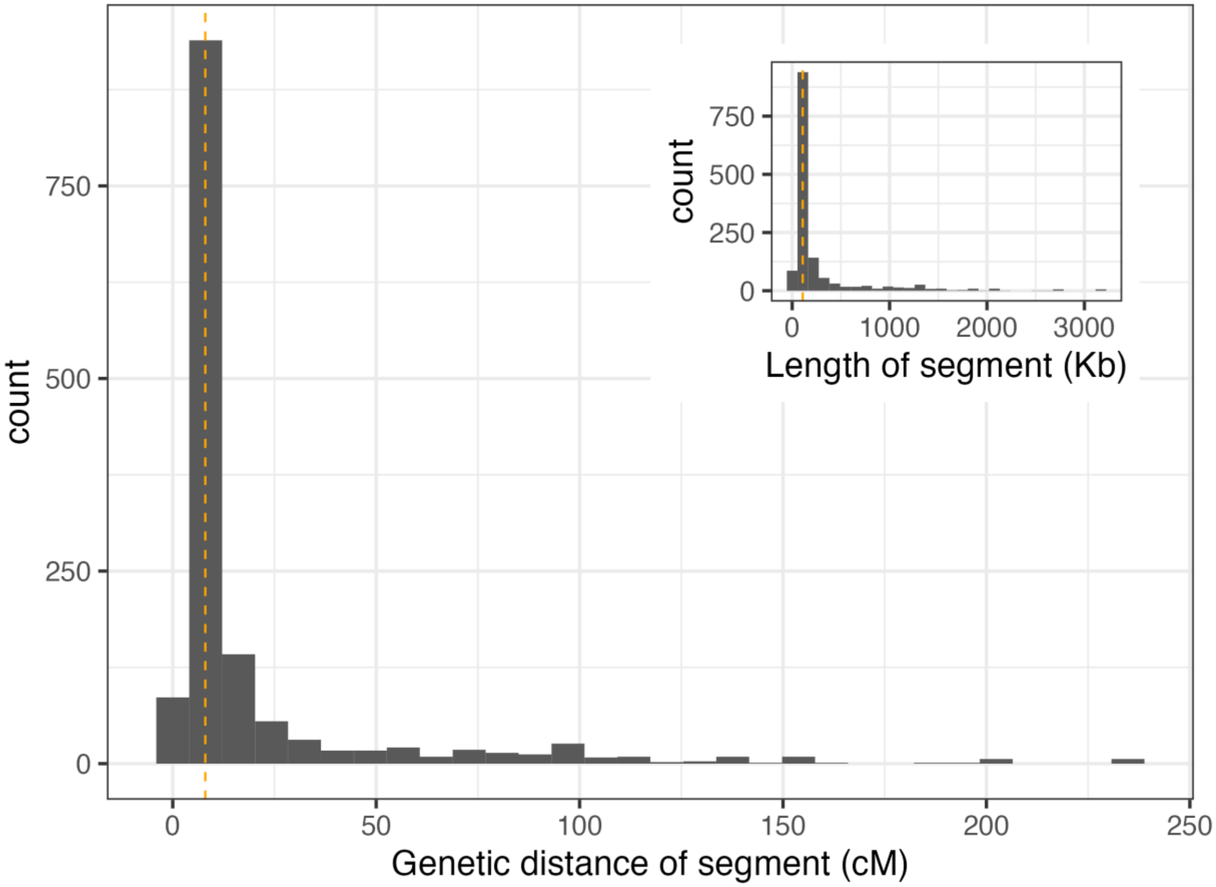


**Supplementary Figure 11. Genetic distance and length (inset) of pairwise IBD segments across the 241 *P. falciparum* sequenced genomes.** The genetic distances (measured in centimorgan [cM] are calculated by assuming a constant recombination rate across the *P. falciparum* genome (13.5Kb/cM). The x-axis represents the length of IBD genomic segments in cent Morgan (cM) and the y-axis represent their frequency across all samples. The vertical dashed red line represents the median genetic distance, equivalent to 8 cM, which corresponds to approximately to six generations (range= 3-239cM).

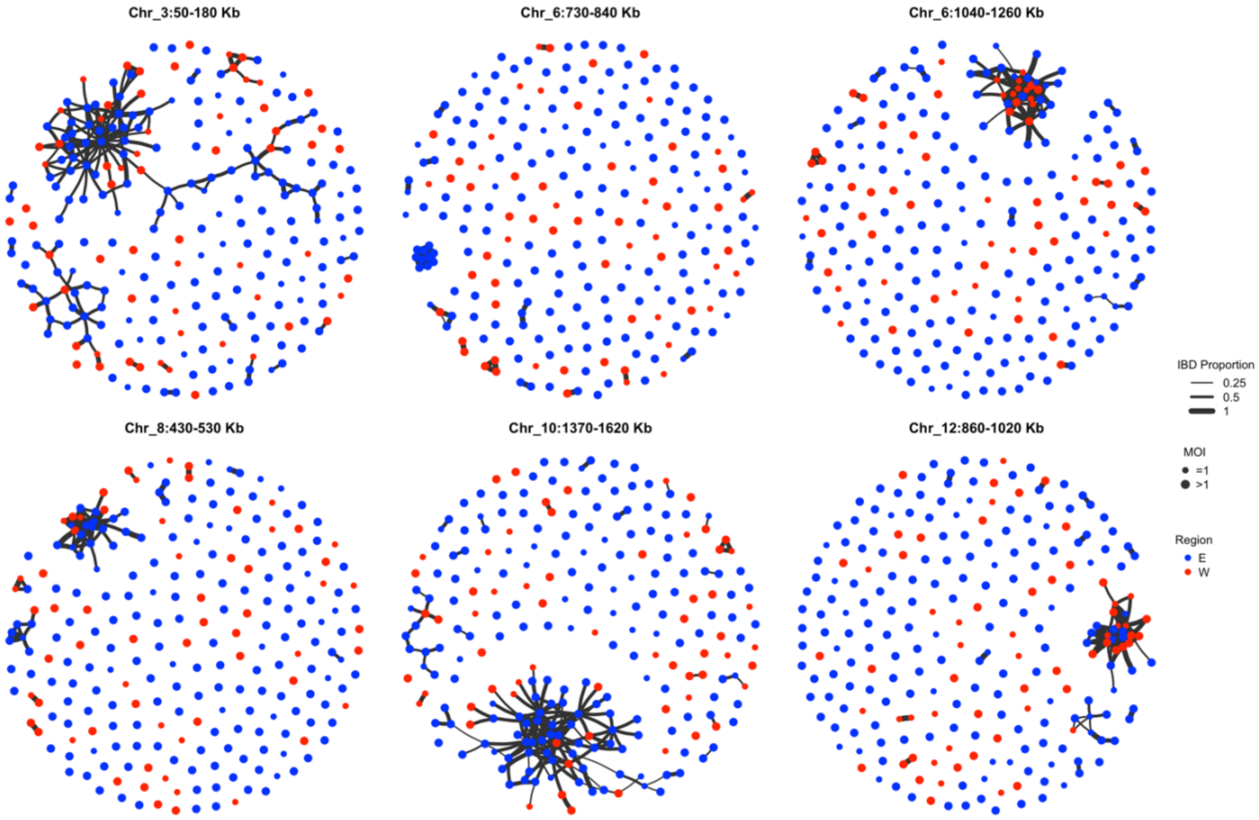


**Supplementary Figure 12. IBD pattern among samples in regions identified under selection using $X_{iR}$ statistics**. Proportion of IBD sharing is calculated for the specified chromosomal regions above each subgraph. Only edges with IBD sharing >= 25% are included. Blue dots represent samples from the Eastern provinces (Luapula, Northern, Muchinga, Eastern). Red dots represent samples from the Western provinces (North-Western, Copperbelt, Western, Central, Lusaka, Southern). Size of the dots represents the mixed infectious status (MOI) of the sample.

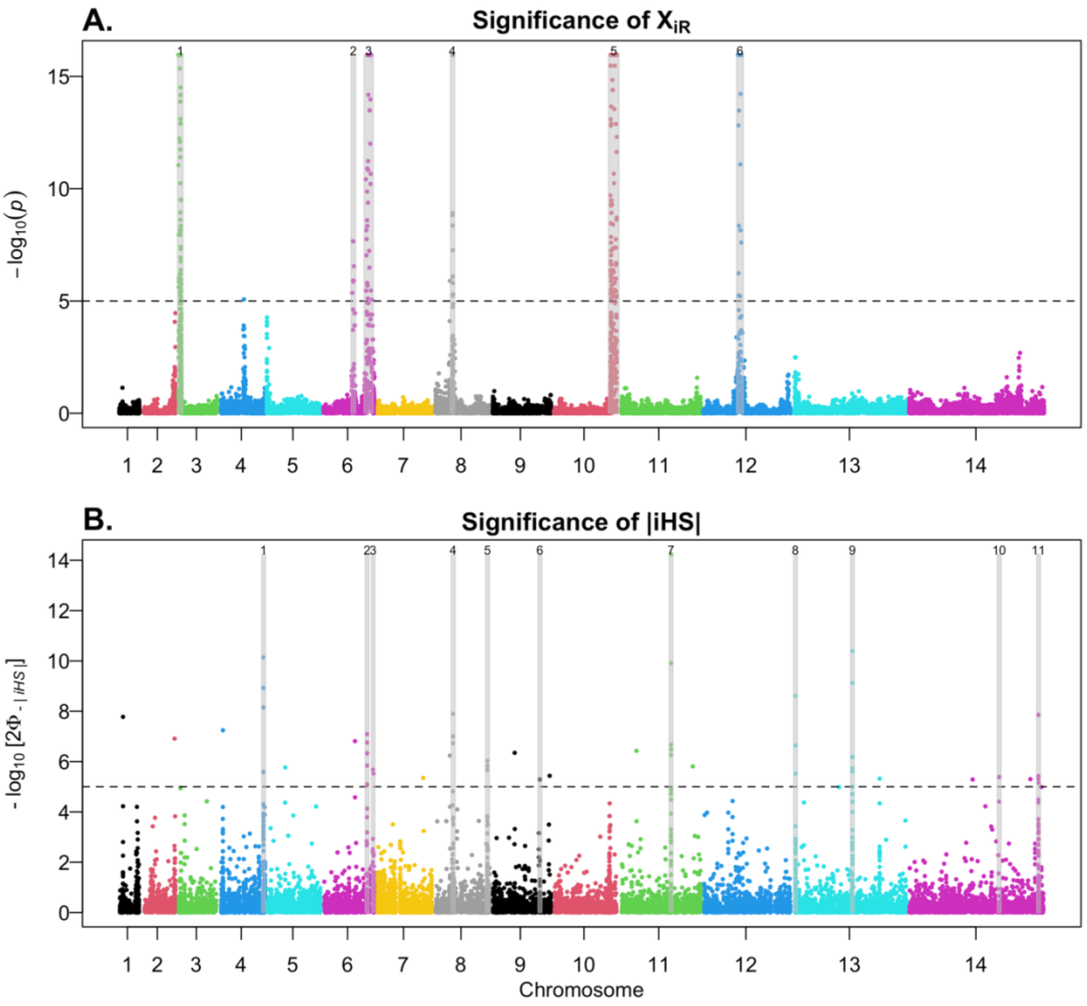


**Supplementary Figure 13**. **Comparison of significantly selected SNPs and regions using $X_{iR}$ vs. Integrated Haplotype Score (iHS) across 14 *P. falciparum* chromosomes.** The graph shows -$\log_{10}$ transformed p-values per biallelic SNPs. The shaded areas represent regions with at least two significantly selected SNPs within a window size of 50Kb. Overlapping areas are joined together. Note that $X_{iR}$ analyses include all 241 samples **(A),** while iHS is performed on the 50 monogenomic samples only **(B).** The plot was generated using the R package rehh.

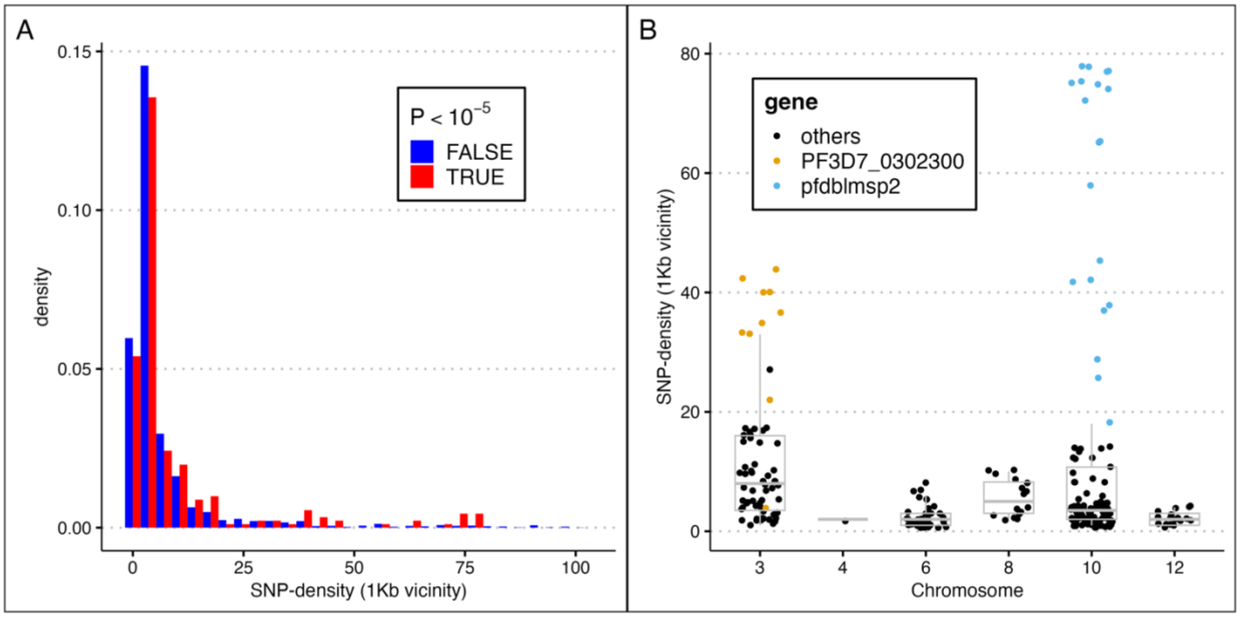


**Supplementary Figure 14. SNP density patterns of different significance status, chromosomes, and genes. (A)** Comparison of SNP-density distributions, calculated within 1 kb vicinity of the focal SNP, between non-significant ($P > 10^{-5}$) and significant sites ($P < 10^{-5}$). **(B)** Distributions of SNP-density on different chromosomes.
