# Supplementary material for: Genomics reveals heterogeneous Plasmodium falciparum transmission and selection signals in Zambia
Source: Commun Med (Lond). 2024 Apr 6;4:67. doi: 10.1038/s43856-024-00498-8 (PMC10998850; doi:10.1038/s43856-024-00498-8)
Supplement: Supplementary file 3 — Description of Additional Supplementary Files. [file 43856_2024_498_MOESM3_ESM.pdf]

Description of Additional Supplementary Files

File name: Supplementary Data 1
Description: Sample description. The table describes 241 P. falciparum WGS sample successfully sequenced (defined as >50% of *P. falciparum* genome with > 5X read coverage) from Zambia, sample origin (province, district and ward levels), cluster ID (smallest geographical unit used in the MIS 2018), cluster coordinates, FWS value and their polygenomic status (i.e. FWS < 0.95 classified as polygenomic and FWS >0.95 classified as monogenomic).

File name: Supplementary Data 2
Description: Polygenomic infection and parasite prevalence data at the cluster level across Zambia. The table describes the number of P. falciparum WGS samples retained from Zambia per geographic cluster (smallest geographic unit according to the MIS 2018 data collection). Percentage of polygenomic infections (number of samples harboring more than one distinct parasite genome (FWS <0.95) divided by total number of samples sequenced per province X100).

File name: Supplementary Data 3
Description: Polygenomic infection and parasite prevalence data at the provincial level. The table describes the number of *P. falciparum* WGS samples retained from Zambia per province (Central, Lusaka and Southern provinces are excluded due to the small sample size, see Methods). Percentage of polygenomic infections (number of samples harboring more than one distinct parasite (FWS <0.95) divided by total number of samples sequenced per province X100).

File name: Supplementary Data 4
Description: Pairwise population differentiation among P*. falciparum* populations across seven provinces in Zambia. Numbers in the upper right indicate pairwise genetic differentiation (FST) values calculated on the genome-wide SNP data set.

File name: Supplementary Data 5
Description: List of the significant selected SNPs across six regions in the chromosomes 3, 6, 8 10 and 12 of the *P. falciparum* genome. This table contains the significant SNPs (snp_id), location of the SNP, XiR, P value and -log10 transformed P-value, and their annotations (original EFFtag, function, Codon changes, amino acid change, gene names, exon number, etc.)

File name: Supplementary Data 6
Description: Range information of significant regions. This table contains the coordinates and SNP statistics of the *P. falciparum* genomic regions with at least two significantly selected SNPs within a window size of 50Kb.

File name: Supplementary Data 7
Description: List of *P. falciparum* genome samples downloaded from the Pf3k database and included for the continental analysis in this study. Sample ID, metadata and sequencing metrics for 714 *P. falciparum* genomes from four African countries (Democratic Republic of Congo, Ghana, Guinea, and Malawi) included in the continental analysis are reported.

File name: Supplementary Data 8
Description: **List of *P. falciparum* genome samples downloaded from SRA originated from Tanzania and included for the continental analysis in this study.** Sample ID, metadata and sequencing metrics for 68 P. falciparum genomes from Tanzania included in the continental analysis are reported.

File name: Supplementary Data 9
Description: List of inferred CNV for significantly selected genes calculated from monogenomic samples. This table contains chromosome, gene location, gene ID, gene length, gene name, gene product description, mean CNV, median CNV, standard deviation of CNV and coefficient of variation of CNV.
