# Supplementary material for: Genomics reveals heterogeneous Plasmodium falciparum transmission and selection signals in Zambia
Source: Commun Med (Lond). 2024 Apr 6;4:67. doi: 10.1038/s43856-024-00498-8 (PMC10998850; doi:10.1038/s43856-024-00498-8)
Supplement: Supplementary file 5 — Reporting Summary [file 43856_2024_498_MOESM5_ESM.pdf]

nature portfolio

Corresponding author(s): Giovanna Carpi, DVM, PhD

Last updated by author(s): Nov 30, 2023

Reporting Summary

Nature Portfolio wishes to improve the reproducibility of the work that we publish. This form provides structure for consistency and transparency in reporting. For further information on Nature Portfolio policies, see our Editorial Policies and the Editorial Policy Checklist.

Statistics

For all statistical analyses, confirm that the following items are present in the figure legend, table legend, main text, or Methods section.

n/a	Confirmed	
☐	☒	The exact sample size (*n*) for each experimental group/condition, given as a discrete number and unit of measurement
☐	☒	A statement on whether measurements were taken from distinct samples or whether the same sample was measured repeatedly
☐	☒	The statistical test(s) used AND whether they are one- or two-sided<br>*Only common tests should be described solely by name; describe more complex techniques in the Methods section.*
☐	☒	A description of all covariates tested
☐	☒	A description of any assumptions or corrections, such as tests of normality and adjustment for multiple comparisons
☐	☒	A full description of the statistical parameters including central tendency (e.g. means) or other basic estimates (e.g. regression coefficient) AND variation (e.g. standard deviation) or associated estimates of uncertainty (e.g. confidence intervals)
☐	☒	For null hypothesis testing, the test statistic (e.g. *F*, *t*, *r*) with confidence intervals, effect sizes, degrees of freedom and *P* value noted<br>*Give P values as exact values whenever suitable.*
☐	☒	For Bayesian analysis, information on the choice of priors and Markov chain Monte Carlo settings
☒	☐	For hierarchical and complex designs, identification of the appropriate level for tests and full reporting of outcomes
☒	☐	Estimates of effect sizes (e.g. Cohen's *d*, Pearson's *r*), indicating how they were calculated

Our web collection on statistics for biologists contains articles on many of the points above.

Software and code

Policy information about availability of computer code

Data collection	Raw Fastq files of P. falciparum WGS data from the Pf3k database used for comparative analysis were downloaded from SRA using pysradb: https://github.com/saketkc/pysradb
Data analysis	Mixed infections were calculated using the R package moimix v.2.9: https://github.com/bahlolab/moimix<br>IBD relatedness and selection were calculated using isoRelate software: https://github.com/bahlolab/isoRelate.<br>Network were visualized with the R package igraph.<br>Custom analysis codes are available from: https://github.com/giocarpi/Pf_wgs_Zambia.

For manuscripts utilizing custom algorithms or software that are central to the research but not yet described in published literature, software must be made available to editors and reviewers. We strongly encourage code deposition in a community repository (e.g. GitHub). See the Nature Portfolio guidelines for submitting code & software for further information.

Data

Policy information about availability of data

All manuscripts must include a data availability statement. This statement should provide the following information, where applicable:

- Accession codes, unique identifiers, or web links for publicly available datasets
- A description of any restrictions on data availability
- For clinical datasets or third party data, please ensure that the statement adheres to our policy

P. falciparum whole genome sequence data are available in the NCBI Sequence Read Archive under BioProject PRJNA932927. These data include the year of sample collection. No other sample information (i.e., related to the individual who provided the sample) was utilized as per the ethical approval for the study.

Research involving human participants, their data, or biological material

Policy information about studies with human participants or human data. See also policy information about sex, gender (identity/presentation), and sexual orientation and race, ethnicity and racism.

Reporting on sex and gender	Data analyzed were from Plasmodium falciparum parasites collected during the 2018 Zambia Malaria Indicator Survey which used a nationally representative two-stage stratified clustering sampling strategy to obtain samples across Zambia for the estimation of malaria prevalence. No human identifiers were used, including information about sex and gender, as per the study design and ethical approval. The study focuses on parasite populations and uses a subset of infections obtained from DBS malaria positive samples that met the criteria for the study. We do not anticipate any bias among infections from individuals based upon sex and/or gender.
Reporting on race, ethnicity, or other socially relevant groupings	No information about race, ethnicity, or other social grouping was obtained for this study. This study involved analysis of malaria parasite infections collected during the 2018 Zambia Malaria Indicator Survey that used a nationally representative two-stage stratified clustering sampling strategy with approximately 25 respondents per cluster across 179 standard enumeration areas or clusters from the ten provinces in Zambia.
Population characteristics	No human population characteristics were used in the study since the study was about malaria infections collected during the 2018 Zambia Malaria Indicator Survey.
Recruitment	The 2018 Zambia Malaria Indicator Survey included dried blood spot (DBS) samples collected from children under the age of 5 from ten provinces across Zambia, with high transmission provinces oversampled. De-identified DBS samples that tested P. falciparum positive by PET-PCR diagnostic test from the ten provinces were included in the genetic study. The majority of DBS samples collected from three provinces with low malaria transmission (Central, Lusaka and Southern) were negative by PET-PCR as well as by RDT and microscopy limiting the number of samples that could be sequenced from these three provinces.
Ethics oversight	The parents or legal guardians provided parental permission for study participants and this study was conducted with the approval of the Biomedical Research Ethics Committee from the University of Zambia (Ref 011-02-18) and from the Zambian National Health Research Authority. All biospecimens and data were de-identified.

Note that full information on the approval of the study protocol must also be provided in the manuscript.

Field-specific reporting

Please select the one below that is the best fit for your research. If you are not sure, read the appropriate sections before making your selection.

☐ Life sciences ☐ Behavioural & social sciences ☒ Ecological, evolutionary & environmental sciences

For a reference copy of the document with all sections, see nature.com/documents/nr-reporting-summary-flat.pdf

Ecological, evolutionary & environmental sciences study design

All studies must disclose on these points even when the disclosure is negative.

Study description	This study involves the use of dried blood spots samples collected from children as part of the 2018 Zambia National Malaria Indicator Survey to determine the level of mixed infections, and genetic relatedness and selection of the parasites from these infections across Zambia. To further contextualize the parasite genomes sampled in Zambia within Africa, WGS data for 781 P. falciparum samples representing 5 countries (Democratic Republic of the Congo, Ghana, Guinea, Malawi, and Tanzania) from the MalariaGEN Pf3k database were included and analyzed for comparison.
Research sample	Dried blood spot samples were collected during the 2018 Zambia Malaria Indicator Survey and were obtained from children under the age of 5 from ten provinces across Zambia, with high transmission provinces oversampled. The rationale was to obtain spatially representative malaria infections across Zambia to understand transmission dynamics, parasite relatedness and selection at the provincial and cluster levels. Dried blood samples are relatively non-invasive, taken at the time of diagnosis, and provide material required for genetic analysis of the malaria parasites from those infections. All samples were collected with ethical approval by the Biomedical Research Ethics Committee from the University of Zambia (Ref 011-02-18) and from the Zambian National Health Research Authority.

Sampling strategy	De-identified DBS samples that tested P. falciparum positive by PET-PCR diagnostic test from the ten provinces were included in the genetic study. The majority of DBS samples collected from three provinces with low malaria transmission were negative by PET-PCR as well as by RDT and microscopy limiting the number of samples that could be sequenced from these three provinces. At least 30 samples were obtained for each of the well-represented seven provinces for P. falciparum whole genome sequencing, a number of samples that has been previously shown to be appropriate for population genomic study.
Data collection	Field teams working with the Zambia National Malaria Control Program collected dried blood spot samples during the 2018 Zambia Malaria Indicator Survey which used a nationally representative two-stage stratified clustering sampling strategy with approximately 25 respondents per cluster across 179 standard enumeration areas from the ten provinces in Zambia. For additional details: https://www.path.org/resources/zambia-natl-malaria-indicator-survey-mis-2018/).
Timing and spatial scale	The 2018 Zambia Malaria Indicator Survey was timed to coincide with the end of the malaria transmission season, from mid-April to late May 2018, which corresponds to the latter portion of the rainy season. This timing is standard practice for MISs in Zambia since 2006.
Data exclusions	Data were excluded if they did not meet the inclusion criteria in terms of genome coverage and sequencing depth.
Reproducibility	A small subset of samples (genomic DNA libraries, ~5%) were re-capture and re-sequenced to evaluate the reproducibility of capture efficacy and sequencing depth of P. falciparum genomes. The data from the different capture and sequencing experiments of the same samples were generally concordant in terms of P. falciparum capture efficacy and sequencing depth.<br><br>Analysis codes are available from: https://github.com/giocarpi/Pf_wgs_Zambia.
Randomization	Randomization was not relevant to the study, as we were evaluating malaria natural infections at the provincial and cluster level for genetic metrics. All samples that met the study criteria (P. falciparum malaria positive DBS samples) were subjected to P. falciparum multiplexed genome capture and sequencing, and samples chosen for downstream genetic analyses were based on inclusion criteria in terms of genome coverage and sequencing depth, and thus randomization was not applicable.
Blinding	Blinding was not relevant to the study

Did the study involve field work? ☐ Yes ☒ No

Reporting for specific materials, systems and methods

We require information from authors about some types of materials, experimental systems and methods used in many studies. Here, indicate whether each material, system or method listed is relevant to your study. If you are not sure if a list item applies to your research, read the appropriate section before selecting a response.

Materials & experimental systems

n/a	Involved in the study
☒	☐ Antibodies
☒	☐ Eukaryotic cell lines
☒	☐ Palaeontology and archaeology
☒	☐ Animals and other organisms
☒	☐ Clinical data
☒	☐ Dual use research of concern
☒	☐ Plants

Methods

n/a	Involved in the study
☒	☐ ChIP-seq
☒	☐ Flow cytometry
☒	☐ MRI-based neuroimaging
